# Navigating the medical physics education and training landscape

**DOI:** 10.1002/acm2.12202

**Published:** 2017-11-10

**Authors:** Brian Loughery, George Starkschall, Kristi Hendrickson, Joann Prisciandaro, Brenda Clark, Gary Fullerton, Geoffrey Ibbott, Edward Jackson, Jay Burmeister

**Affiliations:** ^1^ Karmanos Cancer Center Wayne State University Oncology Detroit MI USA; ^2^ The University of Texas MD Anderson Cancer Center Radiation Physics Houston TX USA; ^3^ University of Washington Radiation Oncology Seattle WA USA; ^4^ University of Michigan Radiation Oncology Ann Arbor MI USA; ^5^ University of Ottawa Radiology Ottawa ON Canada; ^6^ University of Texas Health Science Center Radiology San Antonio TX USA; ^7^ University of Wisconsin Medical Physics Madison MI USA

**Keywords:** ABR certification, DMP, education and training, graduate program, MedPhys match, residency

## Abstract

**Purpose:**

The education and training landscape has been profoundly reshaped by the ABR 2012/2014 initiative and the MedPhys Match. This work quantifies these changes and summarizes available reports, surveys, and statistics on education and training.

**Methods:**

We evaluate data from CAMPEP‐accredited program websites, annual CAMPEP graduate and residency program reports, and surveys on the MedPhys Match and Professional Doctorate degree (DMP).

**Results:**

From 2009–2015, the number of graduates from CAMPEP‐accredited graduate programs rose from 210 to 332, while CAMPEP‐accredited residency positions rose from 60 to 134. We estimate that approximately 60% of graduates of CAMPEP‐accredited graduate programs intend to enter clinical practice, however, only 36% of graduates were successful in acquiring a residency position in 2015. The maximum residency placement percentage for a graduate program is 70%, while the median for all programs is only 22%. Overall residency placement percentage for CAMPEP‐accredited program graduates from 2011–2015 was approximately 38% and 25% for those with a PhD and MS, respectively. The disparity between the number of clinically oriented graduates and available residency positions is perceived as a significant problem by over 70% of MedPhys Match participants responding to a post‐match survey. Approximately 32% of these respondents indicated that prior knowledge of this situation would have changed their decision to pursue graduate education in medical physics.

**Conclusion:**

These data reveal a substantial disparity between the number of residency training positions and graduate students interested in these positions, and a substantial variability in residency placement percentage across graduate programs. Comprehensive data regarding current and projected supply and demand within the medical physics workforce are needed for perspective on these numbers. While the long‐term effects of changes in the education and training infrastructure are still unclear, available survey data suggest that these changes could negatively affect potential entrants to the profession.

## 1 INTRODUCTION

The past decade has been a period of unprecedented change in medical physics education and training. This period of change followed the widespread implementation of specialized, physics‐intensive procedures such as image‐guided radiation therapy and intensity‐modulated radiation therapy, contributing to a demand for medical physics graduates that was much higher than supply.^1^ In a time of increased demand on the quantity of qualified medical physicists required to handle these new technologically complex tasks, an accompanying concern regarding the quality of new entrants to the profession emerged. This concern was, at least in part, spurred by reports^2^, ^3^ of relatively low pass rates (53%) on the Part 3 (oral) component of the American Board of Radiology (ABR) exam (www.theabr.org/ic-rp-landing).

**Figure 1:**
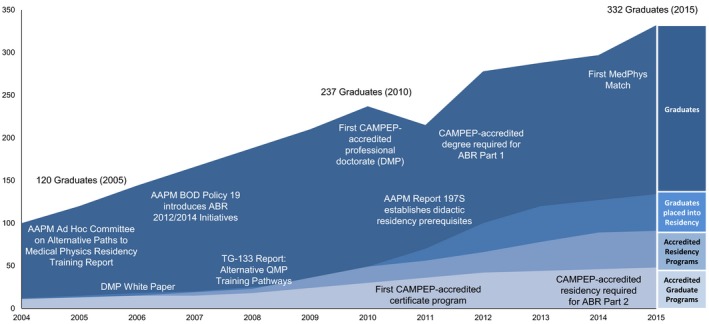
Timeline of major events influencing medical physics education and training along with CAMPEP‐accredited graduate and CAMPEP‐accredited residency enrollment over this time period. Note: some unavailable data interpolated (graduates 2006–2008; graduates placed into residency 2005–2008, 2012, 2014)

A proposed solution was published in a 2000 *Medical Physics* Point/Counterpoint article suggesting that graduation from an accredited graduate or residency program should be a prerequisite for board certification.^4^ While the clinical training provided in an accredited medical physics residency program was commonly accepted as the mechanism to improve the quality and uniformity of clinical training, at the time of publication of the article, only 11 programs (seven graduate programs and four residency programs) were accredited by the Commission on Accreditation of Medical Physics Education Programs, Inc. (CAMPEP).^5^, ^6^ In 2007, the ABR Board of Trustees announced a new requirement for CAMPEP‐accredited clinical training that would be phased in over the time period between 2012 and 2014. The new requirement was in response to the American Association of Physicists in Medicine (AAPM) Board of Directors (BOD) Professional Policy 19, which stated that “graduation from an accredited clinical residency program should be a requirement for qualifying for board certification.” 1 During the “phase‐in” period, the creation of a sufficient number of accredited clinical residency positions was recognized as a significant issue;^1^ consequently the ABR 2012/2014 initiative had a major impact on both the number of accredited graduate programs^6^ and residency programs^5^ (Fig. 1). Beginning 2014, trainees were required to have completed a CAMPEP‐accredited residency program after receiving a graduate degree to become eligible for Part 2 of the ABR certification exam. (Part 1 of the ABR exam can be taken any time after enrollment in a CAMPEP‐accredited medical physics graduate, certificate, or residency program.)

In 2008, AAPM Task Group 133 published the report “Alternative Clinical Training Pathways for Medical Physicists.”^1^ The focus of this report was to describe different training pathways to achieve clinical competency and to outline potential mechanisms for the creation of a suitable number of clinical residency positions. In doing so, this report introduced two initiatives that would become accredited alternative training procedures: a professional degree, the Doctorate in Medical Physics (DMP), which contains both didactic and clinical training, and the certificate program, which provides core didactic elements of a graduate degree in medical physics for students with a PhD from a related outside field. The potential impact of the DMP was discussed in a white paper submitted in 2008 by the AAPM Working Group on a Professional Doctorate for Medical Physics.^7^ Controversy surrounding the creation of this new degree was summarized in a Point/Counterpoint article published in *Medical Physics* in 2008.^8^ The first DMP program was accredited by CAMPEP in 2010, and a total of four such programs have been accredited as of June 2017.^9^ Furthermore, the models presented in TG‐133 suggested the concept of what has become the CAMPEP‐accredited certificate program, although the report does not explicitly mention the concept of a program to provide appropriate didactic education for PhD holders in disciplines other than medical physics. AAPM Report 197S, published in 2011, describes the essential didactic elements of medical physics graduate training for those entering through this alternative pathway.^10^ The first certificate program was accredited by CAMPEP in 2011, and as of March 2017 there were 24 such programs.^11^

In contrast to the situation 10 yr ago, the supply of graduates of CAMPEP‐accredited graduate programs is now greater than the clinical demand.^12^ While the number of accredited residency positions has increased dramatically in response to the new ABR exam eligibility requirements (Fig. 1, Table 1), so has the number of graduates from accredited graduate programs.^13^ There is a significant mismatch between the number of clinically inclined graduates seeking residency positions and the number of positions available. Few question the need for a standardized didactic and clinical training structure, but one might question the process by which we, as a profession, have implemented this standardization. The long‐term consequences are still unclear. Collection and analysis of comprehensive data surrounding these issues is required to begin to evaluate their effects, and this effort is now underway by the combined efforts of the AAPM, CAMPEP, the Society of Directors of Academic Medical Physics Programs (SDAMPP), and the ABR.

**Table 1:** Condensed statistics from CAMPEP annual reports,^15^, ^16^, ^17^, ^18^, ^19^, ^20^, ^26^, ^34^ lists,^5^ and surveys.^14^ Source data for these reports were used to track matriculation. Missing data (–) are unavailable due to lack of collection

Education and training statistics	2009	2010	2011	2012	2013	2014	2015	2016
CAMPEP‐accredited graduate programs	24	30	36	40	44	46	50	52
DMP programs	–	1	1	1	1	2	3	4
Matriculants								
MS	–	–	175	202	187	192	167	196
PhD	–	–	83	103	97	122	114	117
DMP	–	–	–	6	5	10	13	12
Total (w/o Certificate)	196	276	258	311	289	324	294	325
Certificate	–	–	–	14	24	27	79	66
Total	196	276	258	325	313	351	373	391
Graduates								
MS	147	168	148	198	162	164	202	139
PhD	63	69	67	80	113	107	99	89
DMP	–	–	–	–	4	5	6	6
Total (w/o Certificate)	210	237	215	278	279	276	307	234
Certificate	–	–	–	–	9	21	25	24
Total	210	237	215	278	288	297	332	258
CAMPEP‐accredited residency programs	49	53	66	78	89	91	100	107
Incoming residents from CAMPEP‐accredited graduate programs								
MS	17	23	34	45	41	45	56	47
PhD	13	26	28	30	32	38	50	41
Certificate	–	–	–	–	7	10	13	12
Total	30	49	62	75	80	93	120	100
Incoming residents	60	61	70	–	120	140	134	144
Outgoing residents	42	45	53	–	100	103	121	138
Therapy Residents from CAMPEP‐accredited graduate and certificate programs	25	42	56	68	72	81	103	86
Imaging Residents from CAMPEP‐accredited graduate and certificate programs	5	7	6	7	8	12	17	14
Residency placement								
MS	12%	14%	23%	23%	25%	27%	28%	34%
PhD	21%	38%	42%	38%	28%	36%	51%	46%
Certificate	–	–	–	–	78%	48%	52%	50%
Total	14%	21%	29%	27%	28%	31%	36%	39%

The purpose of this work is to summarize the relevant statistics and reports addressing the recent changes in the medical physics education and training landscape and to investigate their effects on various aspects of medical physics education, training and clinical practice. Specifically, we present the current statistics regarding medical physics graduate student matriculation, degree types, graduation rates and demographics of initial placement, with emphasis on residency placement. Additionally, we present these statistics in the form of trends in supply and demand of graduates of CAMPEP‐accredited programs, the state of the conventional residency pathway, and modern alternatives to conventional residency for clinically oriented graduates. We also suggest standardization of data to clearly communicate this information to prospective students interested in the field of medical physics. We hope the data presented here will help elucidate the current and potential future effects of these recent developments on the medical physics education and training infrastructure and on the profession as a whole.

## 2 METHODS

Data were acquired from annually released CAMPEP reports on graduate^13^, ^14^, ^15^, ^16^ and residency programs,^17^, ^18^, ^19^, ^20^ available at http://www.campep.org/ and http://www.sdampp.org/resources.php. These data are presented in Table 1. Additional data were acquired from four surveys: a 2009 SDAMPP survey on the DMP,^21^ a survey of the 2016 SDAMPP annual meeting on medical physics education and training,^22^ the 2017 CAMPEP Residency Program Director Survey,^23^ and a survey of applicants and program directors registered for the MedPhys Match (MPM) during its inaugural 2 yr (2015–2016).^24^, ^25^

In Table 1, the data for accredited graduate programs, graduates, incoming residents with degrees from CAMPEP‐accredited programs, and residency placement were drawn from annual CAMPEP graduate program reports^13^, ^15^, ^16^, ^26^ and CAMPEP/SDAMPP survey results.^14^ These annual CAMPEP reports do not list degree‐specific matriculation data, so we acquired this information from the source data used to create these reports (personal communication with B. Clark, October 1, 2015). The numbers of accredited residency and graduate programs were accumulated from the CAMPEP website in March, 2017.^5^, ^6^ Data on total incoming residents and outgoing residents were found in annual CAMPEP residency program reports^17^, ^18^, ^19^, ^20^ and their source data (personal communication with G. Starkschall, September 13, 2017); however, there was no such report in 2012. MPM data (Table 2) from 2015–2017 were obtained from the National Matching Services website,^27^ available at https://www.natmatch.com/medphys/aboutstats.html, and the post‐MPM news bulletins published by AAPM,^28^, ^29^ available at https://www.aapm.org/pubs/newsletter/default.asp. Since roughly 20% of residency positions were filled outside the MPM from 2015‐2016,^19^ we were unable to obtain complete data on residency placement and demographics from these sources.

**Table 2:** MedPhys Match statistics, condensed from the National Matching Services website.^25^

MedPhys Match statistics 2015–2017			
Year	2015	2016	2017
MPM applicants			
Registrants	402	331	291
Submitted ranks	280	209	224
Ranked applicants^a^	185	157	174
Positions filled	108	106	107
Percentage of applicants who matched			
Registrants	27%	32%	37%
Submitted ranks	39%	51%	48%
Ranked applicants^a^	58%	68%	62%

a Ranked applicants are those ranked by at least one residency program.

To corroborate and elaborate upon the above data, statistics from each CAMPEP‐accredited graduate and residency program were acquired from every individual program website.^30^ Per CAMPEP Graduate Standard 2.10,^31^ accredited programs must “publicly describe the program and the achievements of its graduates and students, preferably through a publicly accessible web site.” This information is to be updated annually, and must include admissions statistics for each degree program. In addition, information on the destinations of graduates must be provided, specifically regarding residency and industry positions.^31^ A template is provided to programs at http://www.campep.org/GraduateProgramSampleDisclosureStatement.pdf as a courtesy, but programs are free to present the data as they choose, which has led to inconsistencies in the way these data are presented. The vast majority of programs binned initial placement of their graduates into residency, industry, advanced degree, and either clinical or academic positions. Placements into research or postdoctoral positions were allocated to “academic” for this report. Those few who were counted as both “academic” and “clinical” were also considered “academic.” CAMPEP recently began providing comprehensive program‐to‐program statistics, collected as survey data for their annual graduate and residency program reports, at http://www.campep.org/PublicDisclosure.asp, though data is currently available for 2016 only.^32^

CAMPEP‐accredited residency programs are similarly required by CAMPEP Residency Standard 2.10^33^ to post data on admissions and placement of graduates from residency into the field, specifically mentioning clinical, academic and industry placement. A template is provided for residency programs as well at http://www.campep.org/ResidencyProgramSampleDisclosureStatement.pdf, and the vast majority of residency programs adhere to this template. Many residency programs also post data on the passing rates of their graduated residents on Part 2 of the ABR Initial Certification exam, but these data was not collected for our study.

As of the date of acquisition of website data, March 31, 2017, several graduate and residency program websites did not have visible links to these statistics on their main pages. A clearly labeled link to the location of program statistics is beneficial for students and evaluators. Eight program webpages did not provide sufficient data for this study: five are so new that they have not posted a graduate, two provided no data on their websites despite being accredited for several years, and one did not bin its residency placements separately from its clinical placements, prohibiting analysis of its data. Admissions, graduation, and graduate placement data from the remaining 44 programs were analyzed.

Website data were further used to verify the accuracy of CAMPEP data in the 2015 reporting year, which was chosen because, at the time of data collection, all but three websites had data for this year. The three deficient websites were accounted for by averaging their output from 2010–2014, which accounted for 4% of the 2015 dataset.

## 3 RESULTS

Table 1 tracks the expansion of CAMPEP‐accredited graduate and residency programs from 2009 to 2015 as reported by CAMPEP.^13^, ^14^, ^15^, ^16^, ^17^, ^18^, ^19^, ^20^ The number of MS, PhD, DMP, and certificate graduates rose by 30% from an average of 221 per year from 2009–2011 to 288 per year from 2012–2014 before peaking at 332 in 2015 (Table 1). It appears that this trend will continue, as the matriculation rate into graduate programs has nearly doubled, rising from 196 in 2009 to 391 in 2016 (Table 1). Caution should be exercised in the interpretation of these data since both the number of accredited programs and the number submitting data to CAMPEP changed over these years. It should also be noted that the CAMPEP graduate reports sort certificate matriculation separately from graduate program matriculation, which is defined as the sum of MS, PhD and DMP matriculants (Table 1). Matriculation into MS programs has not changed significantly over the past 5 yr (Table 1). The mean number of students entering CAMPEP‐accredited MS programs per year is 190 from 2011 to 2015. Over that same period, an average of 44 MS graduates (23%) obtained a residency each year.^15^ The residency placement percentage among graduates from accredited programs rose substantially from 2009 (14%) to 2011 (29%), due primarily to an increase in the number of residency spots and also likely due to the widespread acceptance among students that the clinical residency is the best route of preparation for a career in clinical medical physics. However, it has remained stagnant since, averaging 27% from 2010–2014 before a sharp rise to 36% in 2015. The growth of CAMPEP‐accredited residency programs is also presented in Table 1, with the number of incoming residents rising by 74 from 2009 to 2015. Individual graduate and residency program data were collected from 2015 (webpages incomplete for 2016 as of March 2017) and validated against CAMPEP reports; the sum of all websites agreed with the reports to within 28 graduates (8% error) and four residency positions (3% error). These errors were underestimations, which were expected since several programs have never posted placement statistics or have posted insufficient data for this study.

**Figure 2:**
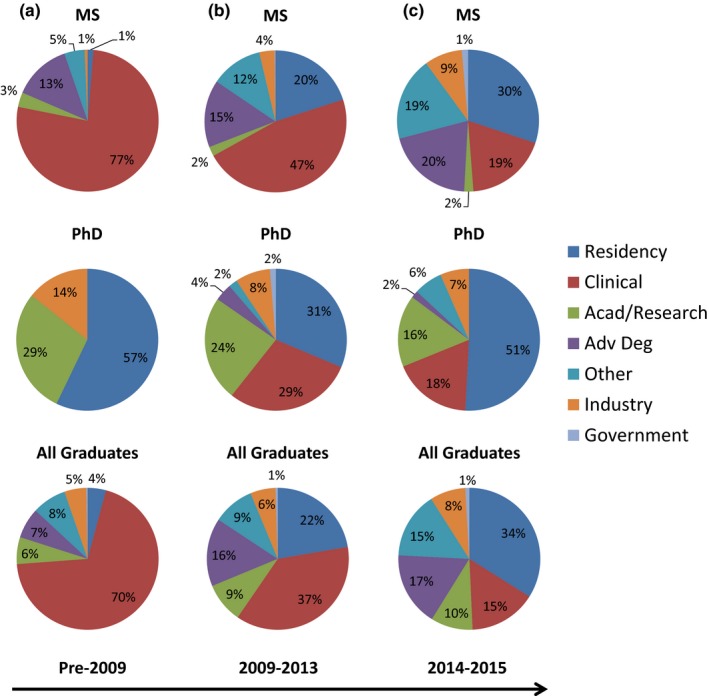
Placement percentages of graduates from various graduate degree programs into professional options, sorted by timeframe (a) Pre‐2009, (b) 2009–2013 and (c) 2014–2015. It should be noted that these data are all from individual program websites. Since many programs do not present data prior to 2009, the first column represents an incomplete picture of placement rates. In addition, many programs do not sort graduate placement by degree, thus the first two rows also represent incomplete data.

Figure 2 shows initial placement of all graduates over three timeframes: prior to 2009, 2009–2013 and 2014‐present, according to the sum of all data found on graduate program websites. The combined rate at which graduates enter clinical work (“Clinical” or “Residency”) fell from 70% (pre‐2009) to 50% (2014–2015), but the percentage of graduates in “Other” (includes those seeking work) rose from 9.0% to 15%. “Advanced Degree” pursuits more than doubled from 7.7% to 17%, while “Industry” and “Academic” categories account for about 9% of the field each, rising from about 6%. The number of academic placements only ranged from 26–29 per year since 2011 with the exception of 2012, which had 19. From 2009–2013, 22% of graduates entered a residency position.

The number of accredited residency programs has more than doubled in recent years,^5^ from 49 in 2009 to 100 in 2015, with the number of accredited residency positions increasing from 60 to 134 over that same period. The total number of 134 residency positions comes from the 2015 CAMPEP residency program report;^19^ unfortunately, this report does not provide the individual numbers of therapy and imaging positions. However, CAMPEP graduate program reports^13^, ^14^, ^15^, ^16^ do separate those graduates who obtained residencies by specialty (therapy and imaging), and only 12% of these entered imaging residencies from 2009–2015. If we extrapolate this percentage to all residency positions (not just those filled by graduates of CAMPEP‐accredited programs), we would estimate 16 imaging residency positions and 118 therapy residency positions offered in 2015. In fact, 17 graduates of CAMPEP‐accredited programs placed into imaging residencies and 103 placed into therapy residencies in 2015. From the CAMPEP residency reports,^19^, ^20^, ^34^ approximately 85% of residency positions were filled by CAMPEP graduates from 2014 to 2016. According to the sum of all program websites, of the 885 total entrants into CAMPEP‐accredited residency programs from 1988 to 2015, 802 (90%) completed residency training. Most residency graduates obtained clinical positions (63%) and academic positions (33%), while the remaining residency graduates entered industry positions (2.3%) or “Other” (1.9%). CAMPEP‐accredited residency program websites were also validated against CAMPEP reports.^20^, ^30^ We found that program websites accounted for 106 residency spots in 2014, compared with 120 graduate placements reported in the 2014 CAMPEP graduate report.^13^ It should be noted that the lack of standardization in reporting makes program website data difficult to interpret. For example, eight residents have been identified as entering industry over the last 2 yr (2013–2014), and four of these were graduates from one residency program, however, it appears from the program website that these graduates are in clinical consulting practice, which most would not consider “industry.” Also, only about half of graduate programs sorted their placement data by degree, which makes it difficult for a prospective student to determine whether data are representative of the degree they seek. Placement from such programs is included in the “All Graduates” row of Fig. 2 but not the rows sorting MS from PhD. Thus, the MS and PhD rows are incomplete data sets.

The percentage of graduates from each CAMPEP‐accredited graduate program that placed into a residency program is presented in Table 3, which compares residency placement percentages in two ways. First, in three timeframes: pre‐2009, 2009–2013 and post‐2014 (Table 3a). Then, by the date in which the programs were accredited (Table 3b). The maximum residency placement percentage, for programs that have placed at least five residents, over all time, is 70%. Among the graduate programs that have *never* placed a resident, the maximum number of graduates is 33. Statistics are generally available beginning only around 2009, even for older programs, so pre‐2009 data from both Table 3 and Fig. 2 are inherently incomplete data sets. Of the 44 CAMPEP‐accredited graduate programs that were included in this study, the median year of CAMPEP accreditation was 2009, and the median residency placement percentage is 22%. All programs that have never placed a graduate into a residency position were accredited after 2009. Our data show that more established programs have placed a higher percentage of their graduates into residency programs than those that have been recently accredited. In the 2017 CAMPEP residency program director survey, over half of responding directors detected moderate‐to‐significant variability in the preparation of admitted residents depending on their particular graduate program.^23^ Since completion of a residency was not required for board certification prior to 2014, many graduates chose permanent clinical employment over available residency positions. Data since 2014 (Table 3) is a true indicator of residency placement percentage of the institution after residency completion became a requirement for board certification. Since the 2014 requirement, the median residency placement rate is 31%, and eight programs have placed no residents.

**Table 3:** The number of CAMPEP‐accredited graduate programs sorted by percentage graduates of placed into residencies, (a) for all CAMPEP graduate programs in the pre‐initiative period (pre‐2009), transitionary period (2009–2013), and post‐initiative period (2014–2015), and (b) by year of CAMPEP‐accreditation. Median residency placement trends upward with time and program age

Residency placement of accredited graduate programs over time and by year of accreditation									
Residency placement percentage	Number of programs							Median	Maximum^a^
	0%	0%–10%	10%–20%	20%–30%	30%–40%	40%+	Total		
(a) All CAMPEP‐accredited graduate programs									
Pre‐2009	13	0	3	0	1	2	19	0%	57%
2009–2013	5	7	4	16	2	8	42	23%	77%
2014–2016	8	2	4	7	4	19	44	31%	93%
(b) CAMPEP graduate programs by year of accreditation									
Accredited 1988–2005	0	1	1	4	3	4	13	30%	70%
2006–2009	0	1	4	2	1	3	11	25%	43%
2010–2011	1	0	4	0	2	3	10	19%	57%
2012–2015	2	2	2	1	1	2	10	11%	41%
Total	3	4	11	7	7	12	44	25%	70%

a Maximum residency placement percentage of programs who have placed > 5 residents.

Residency placement percentages were further sorted by whether the graduate program advertises a clinical component on their webpage or online curriculum. The presence of a hands‐on clinical component within a graduate program could be associated with an increase in the placement of graduates into residency positions. We sorted programs by the presence of a clinical component advertised on their websites or in their posted online curriculum, but we found little difference between those with clinical training components and those without. This could be due to the observation that most graduate programs–32 of 44–include a clinical component, including all programs that have not placed a resident since 2010. Data from a recent survey^24^ of 108 applicants to the MPM and 40 residency program directors support this finding, as program directors ranked “previous clinical experience” as the least important major consideration (other than “other”) for ranking candidates. However, 58% of respondents to the CAMPEP residency program director survey respond that they prefer some informal clinical exposure in their applicants, and 27% prefer formal or extensive clinical exposure.^23^

From CAMPEP reports,^13^, ^14^, ^15^ the residency placement percentage for PhD graduates was 38% from 2011–2015, compared to 25% for MS graduates. The placement percentage for graduates from certificate programs was 56% over this period, though this could be misleading because the sample size for the certificate programs is low in that timeframe (n = 55). Of the 91 CAMPEP‐accredited residency programs in 2015, 21 programs (23%) designated themselves “PhD only” and an additional five gave preference to candidates with a PhD. This is in agreement with the CAMPEP residency program director survey, to which 23 programs (27% of respondents) consider only PhD applicants,^23^ and 65% of respondents indicated that they prefer or require PhD applicants.^23^

Not all graduates from CAMPEP‐accredited programs enter residency positions. Indeed, many graduates are not interested in board‐certified clinical practice and do not apply for residency positions. A valuable metric, particularly for clinically oriented applicants to graduate programs, would be the ratio of graduates entering a residency program to the number attempting to do so. We estimate this metric in Section 4.A below, but data are not currently publicly available. Thus, we are limited to evaluating the ratio of graduates entering a residency to the total number of graduates. Four programs do not have any graduates who entered a residency program between 2011 and 2015, and an additional 11 had fewer than 20% of their graduates enter residency programs. Ten programs, however, have over 50% of their graduates entering a residency program— nearly double the average. The residency placement percentage of a program trends downward with more recent CAMPEP accreditation (Table 3). Eight of the ten programs with very high placement percentages (placement percentage > 50%) had been accredited for at least 5 yr. Seventeen of the 21 programs with a placement percentage greater than 30% had been accredited for at least 5 yr.

Some of the programs with very high placement percentages have small numbers of graduates, thus this statistic is difficult to interpret. Within larger programs, for example, those who graduate at least five students per year, from 2011 to 2015, these statistics become more meaningful. Four programs that have graduated at least 25 students over this timeframe have a placement rate above 40%. These four programs filled 91 of the 430 residency positions (21%) from 2011 to 2015. While 46 programs produce MS graduates, as of March 2017,^15^, ^35^ these four programs accounted for 77 of the 221 MS graduates placed into residency programs (35%) and placed 61% of their MS graduates, which is much higher than the overall placement percentage for PhD graduates (38%). Only 22% of graduates from the remaining 42 accredited MS programs (who educate 75% of all MS graduates) entered a residency program.

Statistics from the 2015 and 2016 MPM^27^, ^28^, ^29^ were compared to CAMPEP residency reports.^18^, ^19^ Residents placed in the MPM accounted for approximately 80% of all spots in 2015. When sorted by degree, the number of residents placed still followed this pattern, but placement percentages were not identical^15^, ^27^, ^28^—MS degree holders fared better within the MPM (33%) than outside (27%), while PhD students fared better outside the MPM (40% in MPM, 51% outside) due in part to residency programs that originated as postdoctoral positions. A recent MPM survey^24^ indicates that the majority of MPM applicants (85% in 2015 and 58% in 2016) consider the current residency placement rate to be a problem for the profession, as opposed to a minority of program directors (40% in 2015 and 33% in 2016). When MPM applicants were asked if prior knowledge of their likelihood of obtaining a residency position would have changed their decision to pursue graduate education in medical physics, a relatively large percentage (38% in 2015 and 25% in 2016) agreed.

## 4 DISCUSSION

Whereas these statistics provide a snapshot of the recent changes in the medical physics education and training landscape, further discussion is necessary to put these data into context, to anticipate how they might change in the future, and to determine whether there are potential unintended consequences associated with these changes.

### 4.A Supply and demand of medical physics trainees

Figure 1 presents a timeline of recent events and associated statistics. The issue of supply and demand within the medical physics profession has been addressed in several publications over the past decade.^1^, ^36^, ^37^ As a result of the ABR 2012/2014 initiative, the large pool of existing unaccredited graduate programs needed to undergo CAMPEP‐accreditation to remain viable, and the number of CAMPEP‐accredited graduate programs subsequently tripled from 15 to 52 from 2007 to 2016.^6^, ^13^ The increase in applications for graduate program accreditation has slowed in recent years (only two new programs in 2016), but the number of matriculating graduate students is at an all‐time high (391, see Table 1), which is resulting in a large number of accredited program graduates (332 in 2015). In 2015 the number of CAMPEP‐accredited residency positions filled by graduates of a CAMPEP‐accredited graduate or certificate program was 120, or 36% of graduates.

One important question surrounding these data is: “how many graduates from CAMPEP‐accredited programs intend to pursue a clinical career?” The 2010 program director survey by CAMPEP^14^ estimated that 62% of graduates pursued clinical work, though it was unclear if this was by choice. In 2014, that same survey estimated that 58% of graduates obtained a clinical position. The 2016 CAMPEP graduate report^15^ estimated that 54% of MS and PhD graduates obtained a residency or junior physics position from 2011 to 2015, but it does not account for those who were unsuccessful in their pursuit. The 2015 CAMPEP residency report^34^ contains placement statistics for the CAMPEP class of 2015 in the MPM. This class includes 169 of the MPM participants, which when divided by the 326 non‐DMP graduates^15^ yields 52%. This estimate does not take into account those who obtained a clinical position prior to the MPM. We conservatively conclude from these data that about 60% of graduate students seek a clinically oriented position after graduation. From 2009 to 2013, 24% of graduates entered a residency position.^13^, ^14^ This indicates that more than half of graduates who desire a residency position will not obtain one in their first year of attempt. This is further supported by the AAPM newsletter review of the inaugural 2015 MPM,^28^ wherein 57% of graduates from the CAMPEP class of 2015 did not match. Thirty‐three percent of these clinically oriented graduates of CAMPEP‐accredited programs were not ranked by any program or withdrew. Of the 91 unmatched graduates, nine were certificate students, 52 held MS degrees, and 30 had PhDs. Half of the unmatched PhD and MS students had withdrawn, compared to a third of certificate students.

While we have significantly expanded the residency training infrastructure, creating a total of approximately 117 positions in radiation oncology and 17 in diagnostic imaging as of 2015,^19^ it is still unclear how many residency positions are really needed. Calibrating the number of residency positions to clinical demand seems to be a reasonable goal.^37^ A mid‐2000's estimate^1^ of the 2020 job market predicted 200–400 new jobs per year. A more recent model by Mills et al.^36^ predicted between 125 and 175 new jobs per year in radiation oncology physics by 2020. Using this prediction, the number of residency positions is potentially on pace to meet demand in 2020, and graduate programs are graduating more than twice the number of students needed to fill these jobs.

Imaging and therapy students face far different realities when attempting to obtain residency positions. Through 2016, website data state that residency programs have graduated a collective 80 residents in imaging, compared to 722 in therapy. This does not account for residency programs in nuclear medicine physics—there were two in 2014.^34^ To resolve the shortage in nuclear medicine physics residency programs, CAMPEP has agreed to allow a “2 + 1” residency program, which allows for imaging physics and nuclear medicine physics residencies to offer an additional twelve months of training of the other discipline to its residents.^34^ Outside of residency program websites, the only data that sort incoming residents into therapy or imaging comes from CAMPEP graduate program reports (Table 1). The 2016 SDAMPP survey respondents estimated^22^ that approximately 130 therapy residency positions are required per year compared to 30 imaging residency positions. By this estimate, about 15–20 more residency positions are required per discipline, which would represent an increase of over 100% for imaging and roughly 20% for therapy. It should be noted that a large number of respondents to this question indicated that they feel there is insufficient data to support these numbers, further illustrating the need to gather and analyze more data to understand the current and future dynamics of the education, training, and clinical landscapes.

Approximately 98% of residents find employment in the field after graduation from a residency program, though the average time between graduation and employment is unknown. This data point was acquired in June 2017 from residency program websites, which makes it a fluid metric by its very nature. Nevertheless, this appears to indicate that residency trainee production is not higher than clinical demand. One potential unintended consequence of the residency requirement is that, in contrast to the former on‐the‐job training model, residency graduates generally complete their clinical training on a fixed date at the end of the residency training term. With the MPM system now in place and common efforts to make the medical physics residency start date coincide with that of medical residents, the production of newly trained medical physicists will now come mostly in a large annual bolus at the end of each June. Until the market adjusts for this bolus, it may be difficult for residency graduates to obtain their first post‐residency position immediately after completion.

The residency placement data per institution presented here must be interpreted with caution as it does not take into account the career objectives of the graduates from that institution. For example, if most of the graduates from a particular program intend to enter non‐clinical careers and therefore do not apply for residency positions, the placement percentage for that program will obviously be relatively low. The percentage of graduates who obtained a residency position out of those who applied for them would be a more appropriate indicator of the success of a program that is striving to place its graduates into board certified clinical practice. Unfortunately, we do not currently have these data. We therefore encourage the collection of these data in the future.

### 4.B Modern residency pathway

The path to a residency position is now largely facilitated by the MPM. The MPM website^27^ claims residency placement percentages of 39% and 51% for the first 2 yr; however, it excludes those who withdrew from the MPM process, due either to not obtaining an interview, accepting a position outside the MPM, realizing they were not on track to graduate in time for MPM, etc. Those who do not withdraw from the MPM are further filtered as “acceptable” applicants, defined by whether or not they were ranked by at least one residency program and not, perhaps more appropriately, by the fulfillment of didactic requirements. The percentage of matched applicants out of these “acceptable” applicants is published in the AAPM newsletters^28^, ^29^ as 55%–60% for 2015 and 61%–74% for 2016. By this definition, over one‐third of graduates from CAMPEP‐accredited graduate programs were classified as “unacceptable” for clinical training in 2015. It is incumbent upon us as a profession to determine whether these rates are acceptable and how current and potential students will interpret and respond to this reality. For the sake of comparison, the match rate for our American PGY1 (post‐graduate year 1) physician colleagues historically ranges between 92% and 95%.^38^

Many graduate students feel pressured to acquire a PhD to improve their chance of obtaining a residency position.^39^ Thus, many clinically oriented graduate students may currently be pursuing or are considering a research degree that they might not want or need to be competitive for a residency position that prepares them for a purely clinical job. While this situation may be unfortunate for students, it is also disadvantageous for the research infrastructure. In reality, to maintain the current academic population, a single researcher need mentor only one PhD graduate student. Any more PhD graduates than this will not have academic positions unless the number of academic positions—a number which has been stagnant over the past 5 yr—increases. While research skills are certainly beneficial to the clinical medical physicist, PhD‐level research mentoring resources may be better spent on students who intend to enter a career in which these imparted research skills will be utilized to expand the frontiers of science. Using them to provide a ticket to a clinical residency spot for an individual who does not intend to pursue a research career is not the best use of these resources and keeps trainees in the education pipeline (and paying tuition) longer than necessary.

Furthermore, attempting to make the PhD/MPM process fit the traditional MD/match model has consequences that may not yet be fully appreciated. Part of this is due to the fact that the MD degree gives both didactic and clinical training, using the residency for specialty clinical training, whereas the PhD degree gives didactic and research training, using the residency as the source of initial clinical training. Medical school education, unlike PhD education, is a structured process with a clear beginning and end. Since most residency positions begin in July, we are implicitly imposing annual graduation timelines for PhD students that may not be tenable. As a result, residency program directors now must take into consideration the degree completion timeline for PhD candidates who have applied to and/or have been accepted into the residency program. This is not unique to the PhD but is true for any graduate program which has a large and thus potentially variable research timeframe.

While about 60% of residency positions from 2009 to 2013 were filled by PhD degree holders,^17^ incoming residents holding MS degrees from CAMPEP‐accredited programs have outnumbered those with PhD degrees from CAMPEP‐accredited programs every year since 2011 (Table 1). The likelihood for an individual PhD graduate to obtain a CAMPEP‐accredited residency position from 2011 to 2015 was approximately 38%, in comparison to an MS graduate who had approximately a 25% probability. The question for some graduate students might become whether this slight increase in probability is worth several additional years of graduate education. Sixty‐five percent of respondents to the 2016 SDAMPP survey^22^ said they would, if they were a clinically focused graduate student, pursue a PhD solely to improve their chances of obtaining a residency position; this is in spite of 56% of the same group agreeing that this is a misappropriation of medical physics research resources. An alternative strategy for those interested only in a clinical career would be to choose a graduate program that places its MS graduates at a very high rate.

Several editorials by Mills and a reply by Beckham and Jackson^39^, ^40^, ^41^, ^42^ discuss the overpopulation of MS degree enrollees and the unfavorable trends facing holders of the MS degree.^39^ Our data suggest the situation is not necessarily unfavorable for an individual student who applies to specific programs, as Mills^40^ and Beckham^42^ suggest. Forty‐six CAMPEP‐accredited programs^6^ (88%) offer a terminal master's degree that is designed to provide appropriate education for clinical practice. As mentioned above, MS students enrolled in four specific graduate programs have historically had a significantly higher chance (61%) of entering a residency program than their colleagues at the MS programs in the 42 other institutions that offer the degree (22%). Also, they have a much higher placement probability than that for all PhD graduates from CAMPEP programs; however, we are unsure of the percentage of these CAMPEP PhD grads that intend to pursue board certification. These data suggest that the MS degree is still a viable option for clinical practice, with the caveat that the clinically oriented student should carefully evaluate the placement data from institutions to which they apply.^35^

The “alternative pathway” to the profession, as described by TG‐133,^1^ has traditionally represented a mechanism for bringing valuable expertise from other disciplines into the field of medical physics. The certificate program was envisioned as a means to formalize the didactic training for such entrants and AAPM Report #197S recommends six areas of core coursework as a minimum requirement for such programs. While the certificate program has standardized the didactic preparation for the alternative pathway and has been rapidly adopted by academic institutions, we must assure that it continues to serve a valuable function for the profession. CAMPEP allows two courses in the topical areas described by Report #197S to be taken during the residency training. Furthermore, many programs have developed online certificate coursework, which may not be ideal for some of these topical areas. As a result, didactic and clinical preparation in medical physics for those entering medical physics residency from certificate programs is relatively modest in comparison to MS and PhD degree holders in medical physics. This is evident in the CAMPEP residency program director survey, to which 100% of respondents prefer graduates of CAMPEP‐accredited graduate programs over those from CAMPEP‐accredited certificate programs.^23^ In December 2016, the AAPM approved the creation of TG‐298 “Task Group on Alternative Pathway Candidate Education and Training” to address these issues.

### 4.C Alternatives to residency for clinically oriented graduates

Graduates interested in entering clinical practice in medical physics who do not obtain a residency position are faced with either attempting to enter the workforce without board eligibility or finding another job where they can apply their medical physics skills (Fig. 2). The number of graduates in the “Other” category, which includes unemployment, has risen from 9% before 2009 to 15% in 2015. While industry is emerging as a proposed path for those who are unable to obtain a residency position, it is unclear how many positions are available for graduates within this career path. It is also unclear how many graduates entering this career path did so by choice or as an alternative after having not matched into a residency program. From the data collected here, approximately 6% of graduates from CAMPEP‐accredited graduate programs have entered industry positions. It will be interesting to see if that percentage rises in the coming years. There are currently initiatives within the medical physics educational infrastructure to train graduates for, and inform them of, non‐clinical career paths such as in industry and government. This is exemplified by the creation of the AAPM Working Group to Promote Non‐clinical Career Paths. In conjunction, the profession should assess and understand the needs of this job market as well as the numbers of current and future trainees who want to pursue these alternatives.

Many graduates who desire a clinical position and are unable to obtain a residency position perform volunteer clinical work to gain experience. Others are willing to pay for clinical experience. A residency program that charges $31,500 in tuition ($15,750 per year) was accredited by CAMPEP in October, 2016.^43^ Similarly, the viability of the DMP is based on the willingness of students to invest in clinical training that leads to board eligibility. Indeed, a survey of DMP program directors revealed the mean cost of a 4‐yr DMP to be about $70,000 in‐state and $95,000 out‐of‐state.^44^ The DMP was promoted as an alternative to a residency pathway^1^ at a time when there was a shortage of formal clinical training programs. It is unclear whether there remains a shortage of residency positions, and while comprehensive workforce studies have been previously undertaken,^12^, ^36^, ^45^ additional data will be required to answer this question.

We might expect a DMP program to be an attractive option given that it provides guaranteed clinical training and board eligibility since it incorporates the accredited residency training within the degree program. However, the DMP student typically pays tuition for their clinical training, in contrast to the traditional resident who is paid a salary. As a result, the DMP may be perceived as a wise investment for incoming students since it provides accredited residency training experience as required by the ABR, but the DMP may also be seen as a last resort for graduates who cannot obtain a competitive paid residency. The DMP might ultimately be perceived as simply a very expensive way to obtain a residency spot and ABR eligibility. Despite the increased financial burden, it has been suggested that the DMP graduate will reach higher financial status over a 7‐yr plan than other professional doctorates in other fields.^46^

Many have been and are currently considering whether the DMP is beneficial to the profession.^8^, ^46^ It may be a valuable pathway to the creation of a sufficient number of clinical training opportunities to meet clinical demand and a mechanism to produce more well‐rounded entrants by requiring more graduate education than MS degree requirements. One could also argue that it could degrade the foundation of the training pipeline built on the competitive forces that weed out all but the best entrants into the profession, instead allowing those who are willing to pay a very large sum for tuition. As it was previously noted that the path to a residency position is now largely facilitated by the MPM, it should be noted here that DMP programs exist exclusively outside the match.

Nine programs indicated in the 2015 CAMPEP survey that they have created, are in the process of creating, or are considering creating a DMP program. If all of these programs come to fruition and become accredited, we can anticipate approximately 15 additional accredited clinical training positions in radiation oncology and approximately two additional positions in diagnostic imaging. The 2009 SDAMPP survey on the DMP indicated that more respondents felt that the DMP would improve clinical training but degrade both research and the stature/credibility of the profession.^21^ It should be noted that the “students” subgroup was the only group that did not feel that the DMP would degrade the stature/credibility of the profession. While the perceived overall effect of the DMP of all respondents to the survey was negative, 20% of student respondents stated that they would have applied to a DMP instead of an MS program if one had been available. A 2016 SDAMPP survey^22^ posed many questions with regard to the DMP. Seventy‐four percent of respondents indicated that they do not see the DMP as an improvement in medical physics training, with the vast majority of concerns mentioning tuition and the lack of a distinction between DMP and MS degree with a subsequent residency. When asked what a DMP should consist of, only 19% of respondents believed it should be only an MS degree plus a residency. Thus, while standards for accreditation for DMP programs exist already, the majority of respondents to the SDAMPP survey feel that these are not comprehensive enough. The vast majority of respondents (78%) felt that the format of a DMP should be standardized across institutions. It is clear from this report that there is still considerable uncertainty about the DMP among those involved in medical physics education and training. Additional standardization of the DMP degree beyond current accreditation requirements may be helpful in demonstrating its value to the profession.

The number of graduates from CAMPEP‐accredited graduate programs currently outpaces the number of accredited residency spots by a factor of three. This mismatch continues to be a topic of debate.^37^ Increasing the number of residency and/or DMP programs would shift the bottleneck to the transition from these programs to clinical practice. However, those who fail to obtain a job will be left unemployed after four or more years of training, at least two of which cost graduate‐level tuition. Reducing the number of graduate students would shift the bottleneck to incoming undergraduates and alternative pathway transfers who, if accepted, will have a higher chance to obtain certification and employment in medical physics. It could be argued that stronger competition for residency positions results in a stronger crop of clinical trainees—residency programs get to select the “cream of the crop” for their positions. However, it is not necessarily in the best interest of the student, and we should carefully consider the effect on our applicant pool.

At present, we may be deterring prospective medical physicists from this career path due to the significant uncertainty about whether they will have an option for board‐certified clinical practice.^47^ This possibility is strongly suggested by the results of the recent MPM survey which indicated that 72% of MPM applicants who completed the survey consider the residency placement a problem to the profession, and 40% and 25% of respondents in 2015 and 2016, respectively, agreed that they would have reconsidered joining the profession had they known of this issue before entering graduate school.^24^ If the field continues to train such a large number of graduate students with a small but near‐appropriate number of residency and DMP positions, the effects on our applicant pool must be determined. At the very least, prospective students must be provided accurate and easily accessible data.

### 4.D Data presentation

CAMPEP‐accredited graduate programs are required^31^ to post a table of admissions and graduate placement statistics on their program website, but these tables are inconsistent from program to program. This inconsistency makes it difficult for prospective students to compare programs or to even know if the statistics are relevant to their degree or career of interest. Some key issues were apparent while collecting the data for this study. Specifically, it is not clear to what year the data should be attributed since this could be interpreted as either the year of matriculation or the year of graduation. It is not always clear to what degree the data is attributable since some programs do not separate the data by degree, nor is it clear what constitutes an “academic” position, or how many graduates intended to enter a residency but entered a different path because they were unable to obtain one.

We suggest that the meaning of data tables should be standardized to make these data explicit. This standardization could include, for example, graduates posted represent those graduated in that year, positions with multiple components are counted once by largest fractional time, data are sorted by degree obtained, and the number of graduates who applied for residency is presented alongside the number who entered residency. With such standardization, a collection of all website data could be accurately and robustly displayed on a single webpage sorted by program. CAMPEP recently posted the results of the 2016 graduate program survey publicly at http://www.campep.org/PublicDisclosure.asp. Other possible sites are the AAPM student webpage at http://www.aapm.org/students/, which already contains a guide to medical physics education and training for undergraduates,^48^ and the SDAMPP webpage at http://www.sdampp.org/resources.php, which has links to all CAMPEP reports to date. In the absence of such a collection, residency applicants have resorted to organizing their own online surveys of their experiences and have created an interactive map based on these informal results, courtesy of the AAPM Student and Trainee Subcommittee.^49^, ^50^ It is not hard to imagine that a prospective student would look at these data—and at our entire education and training landscape—and see a career path that may not seem worth the level of uncertainty it represents. This would be a serious and negative unintended consequence of the recent changes to the training requirements for ABR certification.

In summary, we, as a profession, should carefully consider the allocation of our training resources. We would be wise to determine approximately how many medical physicists we should be educating and training. Efforts focused on creating an education and training environment that both attracts and cultivates the highest quality trainees will result in the highest quality workforce that will provide the clinical care and create the scientific breakthroughs of the future. We should also allocate research resources toward future researchers–not as a means to improve the chances of graduates to obtain a clinical residency spot, but to drive meaningful scientific advances. We should consider the effects of the current state of medical physics education and training on our future graduate students and trainees. Our students deserve honesty about the current residency and job market and their relative chances of doing what they want to do with a degree from our programs. They also deserve easily accessible information on potential career paths along with data to facilitate their appraisal of the viability of their career plans. Those of us involved in medical physics education and training should carefully consider the recent changes that have taken place and their effects on the future of the profession. We then need purposeful action to drive the education and training landscape to where we would like it to be. We are unlikely to get there by unguided chance.

## 5 CONCLUSION

We present data from publicly available CAMPEP reports, program websites, surveys, and publications to illustrate and to discuss the wide‐ranging changes to the education and training landscape in medical physics over the past decade. Data collected from program webpages correlate well with CAMPEP reports and surveys, though these data could be made more clear and standardized. The rapid proliferation of CAMPEP‐accredited graduate and residency programs, together with the implementation of new requirements for eligibility for board certification, has resulted in significant changes in the education and training pathway. The production rate of accredited graduates increased by over 50% in just 7 yr between 2009 and 2015, and this rate is still increasing, as the 2016 incoming class is the largest ever. While we estimate that approximately 60% of graduates from CAMPEP‐accredited graduate programs would like to enter clinical practice, only approximately 29% obtained a residency between 2011 and 2015. The number of graduates of CAMPEP programs outpaces the number of residency spots by a factor of three, and there is still significant uncertainty in the number of residency positions we need. However, to provide clear data on this in the future, we recommend that survey data requested from graduate programs gather the number of graduates who obtained residency positions and the number who intend to enter board‐certified clinical practice.

The residency placement percentage has risen over the past 5 yr, but large differences exist between accredited graduate programs in their placement of graduates into clinical practice, with percentages correlated with program age and independent of the presence of a clinical component. Only approximately one in four graduates of CAMPEP‐accredited MS programs currently obtain a residency position, a fact which is encouraging some students to pursue a PhD to increase their odds to one in three. An alternative solution may be to enter an MS program with better placement statistics. Thirteen programs have residency placement rates greater than 40% from 2011 to 2015. The other current alternative is to pay tuition for clinical training through a DMP program. The long‐term effects of the implementation of the DMP degree on our profession are still unclear, but survey results from those involved in medical physics education and training indicate that additional standardization of this new degree type would be beneficial in demonstrating its utility to the profession. Those same survey respondents overwhelmingly support the need to collect comprehensive data to better evaluate our current education, training, and clinical needs. We have implemented new education pathways, but it is not yet clear how these might affect our training infrastructure. Finally, we have evidence that these uncertainties could influence prospective entrants to the profession and thus the future quality of our applicant pool.

## CONFLICTS OF INTEREST

The authors have no actual or potential conflicts of interest for the work presented here.
